# Integrating Porphyrinic Metal-Organic Frameworks in Nanofibrous Carrier for Photodynamic Antimicrobial Application

**DOI:** 10.3390/polym13223942

**Published:** 2021-11-15

**Authors:** Huiru Zhang, Zhihao Xu, Ying Mao, Yingjie Zhang, Yan Li, Jihong Lao, Lu Wang

**Affiliations:** 1 Key Laboratory of Textile Science & Technology, Ministry of Education, College of Textiles, Donghua University, Shanghai 201620, China; zhrlzc@163.com (H.Z.); lyabblove@126.com (Z.X.); maoying_dhu@163.com (Y.M.); 15900238890@163.com (Y.Z.); ljh@dhu.edu.cn (J.L.); wanglu@dhu.edu.cn (L.W.); 2 Key Laboratory of Textile Industry for Biomedical Textile Materials and Technology, Donghua University, Shanghai 201620, China

**Keywords:** metal-organic frameworks, photodynamic therapy, antimicrobial nanofibers, wound dressing

## Abstract

The rise and spread of antimicrobial resistance is creating an ever greater challenge in wound management. Nanofibrous membranes (NFMs) incorporated with antibiotics have been widely used to remedy bacterial wound infections owing to their versatile features. However, misuse of antibiotics has resulted in drug resistance, and it remains a significant challenge to achieve both high antibacterial efficiency and without causing bacterial resistance. Here, the ‘MOF-first’ strategy was adopted, the porphyrinic metal-organic frameworks nanoparticles (PCN−224 NPs) were pre-synthesized first, and then the composite antibacterial PCN−224 NPs @ poly (ε-caprolactone) (PM) NFMs were fabricated via a facile co-electrospinning technology. This strategy allows large amounts of effective MOFs to be integrated into nanofibers to effectively eliminate bacteria without bacterial resistance and to realize a relatively fast production rate. Upon visible light (630 nm) irradiation for 30 min, the PM−25 NFMs have the best ^1^O_2_ generation performance, triggering remarkable photodynamic antibacterial effects against both *S. aureus*, *MRSA*, and *E. coli* bacteria with survival rates of 0.13%, 1.91%, and 2.06% respectively. Considering the photodynamic antibacterial performance of the composite nanofibrous membranes functionalized by porphyrinic MOFs, this simple approach may provide a feasible way to use MOF materials and biological materials to construct wound dressing with the versatility to serve as an antibacterial strategy in order to prevent bacterial resistance.

## 1. Introduction

Antibiotic-resistant bacteria (ARB) infection has become a global crisis in wound management, which causes a delay in healing and a corresponding spike in healthcare expenses [1,2,3]. The World Health Organization (WHO) estimates that if no action is taken, the global annual cost of ARB could increase to US$100 trillion and 10 million deaths by 2050, which is far exceeding the number of cancer-caused deaths [4,5,6,7]. To overcome the arduous challenge of ARB infection, organic quaternary ammonium compounds [8,9,10,11], antimicrobial peptides [12,13], metal ions [14,15], and metal oxide nanoparticles (NPs) [16,17] have been extensively applied as alternative bactericidal agents to combining with the wound dressings. However, their existing shortcomings of certain cytotoxicity, material instability, or even increased risk of bacterial resistance in practice cannot be ignored [18,19,20]. Therefore, what is vital to wound management is the development of new therapeutics less prone to resistance.

Photodynamic therapy (PDT), an invasive approach with high spatiotemporal accuracy has become an alternative weapon against ARB [21,22]. During PDT, the photosensitizers (PS) enriched in the wounds can be activated by appropriate light, resulting in producing reactive oxygen species (ROS, i.e., ^1^O_2_, OH, O^2−^, and H_2_O_2_) for sterilization [23,24]. Moreover, it is essential that the bacterial cell damage induced by ROS is non-specific, which is unlikely to result in drug resistance [25]. Unfortunately, there are disadvantages of PS that cannot be ignored, such as poor chemical stability, water-insolubility, and the tendency to self-agglomeration under physiological conditions severely restricts the capability to generate ROS, which hinders the application of PS in wound care [26]. Currently, conjugating PS with carriers has been applied as an effective countermeasure. Metal-organic frameworks (MOFs) have been proven to be a unique nanocarrier for functional agents owing to their extremely high specific surface area, plenty of porous channels, and versatile coordinate sites [27,28,29]. In particular, an excellent synergistic effect on the functionality of the resultant MOFs-PS conjugates was exhibited, the agglomeration and self-quenching of PS have been avoided, and the photodynamic activity has been greatly enhanced [30,31]. Encouraged by synergistic effect, current research is devoted to combining MOFs with a carrier to avoid the drawback of MOFs in pure powder forms that are not easily enriched at the wound site [32].

Electrospun nanofibrous membranes (NFMs) offer remarkable advantages, such as variable pore size, large specific surface area, and oxygen permeability, which promote cell adhesion and rapid proliferation, making them suitable for MOFs immobilization [33]. Electrospinning, as a versatile and affordable technology for the facile and rapid fabrication of ultrafine polymer nanofibers, has been widely used in various fields such as advanced batteries [34], wastewater purification [35,36,37], and biomedicine [38]. Importantly, the features of electrospinning nanofibrous membrane structural stacking are similar to that of natural extracellular matrix, making it suitable for wound-dressing applications. Qian et al. [39] incorporated the ZIF-8 MOFs loaded with the PS (rose bengal, RB) into the electrospun poly (ε-caprolactone) (PCL) nanofibrous matrix via an ‘MOF-first strategy’. Notwithstanding that the instability of ZIF-8 may impair therapeutic effectiveness, the as-prepared dressing still shows a good exploitation potentiality because of the features of NFMs. Until now, there have been many different nanofiber spinning technologies developed, for instance, Forcespinning^®^, a versatile technique for the production of high-throughput polymer nanofibers [40,41]. Co-electrospinning is more suitable and practical for small-scale tests in the lab considering the engineering challenges embracing the scalability of performing methods and the integrated stability between MOFs and fibers are still required to be resolved urgently.

**Scheme 1:**
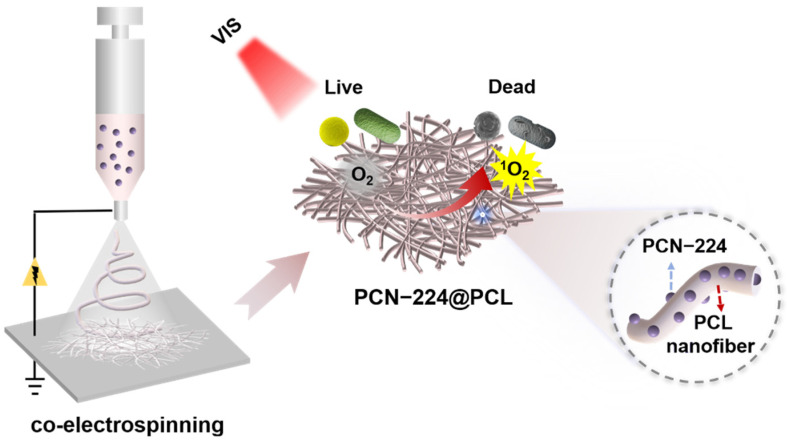
Schematic diagram of the preparation of the PCL nanofibrous membranes containing PCN−224 NPs (PM NFMs) photodynamic antibacterial NFMs.

Here, our conception of the photodynamic antibacterial wound dressings for infection is based on the assessment of two criteria: (1) the inherent biosafety (biodegradable and biocompatible properties) of dressings should be guaranteed, (2) the PS must be non-agglomerated and firmly incorporated with the nanofibers at high content. The first criterion is met by using the Food and Drug Administration (FDA) proven synthetic polymer—PCL [42]. Although natural materials such as chitosan [43], BSA [44], gelatin [45,46], zein [47], and collagen [48] also demonstrate the biodegradable and biocompatible properties, problems persist with their poor electrospinnability, fast degradation rates, and mechanical instability. In addition, PCL would not generate harmful acidic (lactic and glycolic acids) degradation products such as polylactic acid and polyglycolic acids do. In order to meet the second criteria, we used Zr-based porphyrinic MOFs (porous coordination network−224, PCN−224) NPs as PS, which have excellent properties such as easy synthesis, and good biocompatibility [49]. The excellent acid/alkali stability of PCN−224 NPs provides the possibility to incorporate with PCL via the co-electrospinning method (Scheme 1). The process conditions are moderate which can be tolerated by PCL and the high-quality MOFs can be obtained because their nucleation and growth processes can be optimized ex-situ. Consequently, the PCL nanofibrous membranes containing PCN−224 NPs (PM) NFMs serve as an antibacterial wound dressing that can generate ^1^O_2_ under the 630 nm red light irradiation to attack bacteria. Such PM NFMs would be potential candidates to quickly eliminate bacteria at the wound site while preventing the development of ARB.

## 2. Materials and Methods

### 2.1. Materials and Reagents

Zirconyl chloride octahydrate (ZrOCl_2_·8H_2_O, 98%), benzoic acid (≥99.5%), and poly (ε-caprolactone) (PCL, Mn = 80,000) were purchased from Sigma-Aldrich Chemicals (Shanghai, China). Tetrakis (4-carboxyphenyl) porphyrin (TCPP), 97%) and 2-[4-(2-hydroxyethyl)-1-piperazinyl] ethanesulfonic acid (HEPES, 99%) were obtained from TCI (Shanghai) Industrial Development Co. (Shanghai, China). N,N-dimethylformamide (DMF, 99.8%), and 1,3-diphenylisobenzofuran (DPBF, 97%) were bought from J&K Scientific Ltd. (Beijing, China). Chloroform (CHCl_3_, AR), and acetone (C_3_H_6_O, AR), both reagents were provided by China National Pharmaceutical Group Corporation (Shanghai, China). A Cell Counting Kit-8 (CCK-8) was purchased from Yeasen Biotechnology (Shanghai) Co., Ltd. (Shanghai, China). Gram-positive *Staphylococcus aureus* (*S. aureus*, ATCC 29213), Gram-negative *Escherichia coli* (*E. coli*, ATCC 25922), and methicillin-resistant *Staphylococcus aureus* (*MRSA*, ATCC 43300) were obtained from Shanghai Jiachu Biological Engineering Co., Ltd. (Shanghai, China). Mouse fibroblast (L929) was obtained from Shanghai Cell Bank of Chinese Academy of Sciences (Shanghai, China). All chemical reagents were purchased and used directly without further purification.

### 2.2. Synthesis of PCN−224 Nanoparticles (NPs)

The synthetic procedure to obtain PCN−224 NPs was according to the solvothermal method reported in the previously published protocol [50]. Briefly, 10 mL ZrOCl_2_·8H_2_O solution (15 mg/mL, solvent is DMF), 20 mL TCPP solution (2.5 mg/mL, DMF) and 20 mL benzoic acid solution (70 mg/mL, DMF) were dissolved uniformly under ultrasonic shaking, respectively. Then we added them to a 250 mL round bottom flask in sequence and the mixed solution was stirred at 90 °C for 5 h in an oil bath. After the reaction, the compound was allowed to cool to room temperature, the product was collected by centrifugation at 12,000 rpm for 30 min and followed by washing with DMF three times to remove unreacted substances. The final PCN−224 NPs were resuspended in fresh DMF and stored in the dark.

### 2.3. Preparation of the PCL Nanofibrous Membranes Containing PCN−224 Nanoparticles (PM NFMs)

The PM NFMs were fabricated by the co-electrospinning method. Firstly, PCL was dissolved in a mixture solvent of CHCl_3_ and DMF (1:1, *v*/*v*) to get a homogeneous solution with a concentration of 15 wt%. After the PCL was completely dissolved, PCN−224 NPs of different mass ratios were added, and the mixed solution was ultrasonic for 30 min and then placed on a magnetic mixer for 12 h until the PCN−224 NPs were evenly dispersed in the spinning solution. The homogeneous polymer solutions were store in a 10 mL syringe with a 22 G needle, followed by electrospinning at a voltage of 23 kV and feed rate of 0.9 mL/h. The nanofibers were collected on a collector 15 cm away from the tip of the needle. The entire electrospinning process was carried out under the temperature and relative humidity were 22 ± 2 °C and 44 ± 2%, respectively. The obtained the PCL nanofibers containing PCN−224 NPs were denoted as PM NFMs (PM−0, PM−10, PM−25 and PM−40) based on the weight ratios of PCN−224 NPs relative to PCL (0%, 10%, 25% and 40%, *w*/*w*). The PM NFMs were vacuum-dried for 12 h at 30 °C to remove the residual solvent in the fiber membrane and then protected from light.

### 2.4. Detection of ^1^O_2_ Formation

^1^O_2_ detection was carried out by using 1,3-diphenylisobenzofuran (DPBF) as a chemical probe [32,49,51]. Briefly, the PCN−224 NPs were incubated with 3 mL of DMF solution with a concentration of 10 μg/mL DPBF. Then the cuvette was placed under visible light of 630 nm (100 mW/cm^2^) for 10 min, and the absorbance of the DPBF solution at 415 nm was measured every 2 min. Pure DPBF solution was used as a control sample. The test for PM NFMs is the same as PCN−224 NPs, except that the reaction medium was changed from DMF to methanol.

### 2.5. Photodynamic Antibacterial Assay

Gram-negative *E. coli* (ATCC 25922), Gram-positive *S. aureus* (ATCC 29213), and *MRSA* (ATCC 43300) were selected as representative strains to evaluate the photodynamic antibacterial ability of PM NFMs. In short, bacterial strains were cultivated overnight in Luria-Bertani (LB) medium with shaking at 37 °C. Subsequently, the concentration of the bacterial solution was diluted to 10^7^ CFU/mL with PBS buffer (0.1 M, pH = 7.0). Then we drew 100 μL of bacterial solution (10^7^ CFU/mL) to add to the prepared PM NFMs (1 × 1 cm^2^), pre-cultured in the dark for 30 min, and irradiated it under visible light of 630 nm (100 mW/cm^2^) for 30 min. The control sample was incubated in the dark for 60 min. Afterward, 0.9 mL PBS was added to each sample, and the bacteria adhered to the membranes were removed by sonication for 15 min. Then, the bacteria supernatant was 10-fold gradient diluted with PBS, and 100 μL of each dilution gradient was inoculated on LB agar plates. The treated agar plate was placed in a 37 °C constant temperature and humidity incubator for 18–24 h. We counted the number of colonies on the agar plate to evaluate its photodynamic antibacterial performance. This was done for each sample three times.

### 2.6. Cytotoxicity Assay

A standard CCK-8 assay was selected to evaluate the cytotoxicity of PM NFMs. First, L929 cells were cultured in the complete medium containing 90% DMEM, 10% fetal bovine serum, and 1% penicillin-streptomycin solution. Subsequently, the cells were inoculated on 14 mm PM NFMs at an implantation density of 2 × 10^4^ cells per well, the wells without samples were used as controls, and the well plates were placed in the incubator after inoculation was completed. After 24 h incubation, the medium was aspirated from each well and the cells were washed three times with PBS to remove unadhered free cells. Finally, 500 μL of CCK-8 working solution was added to each well under dark conditions, and the absorbance of the supernatant at 450 nm was measured after 3 h of incubation at 37 °C incubators. This was done for each sample three times.

### 2.7. Characterizations

The surface morphological structures of PCN−224 NPs were characterized by field emission-scanning electron microscopy (FE-SEM, SU-8010, Hitachi Ltd., Tokyo, Japan), scanning/transmission electron microscopy (STEM, Talos F200S, Thermo Fisher Scientific, Waltham, MA, USA). *N_2_* adsorption-desorption isotherms were selected to analyze the Brunauer–Emmett–Teller (BET) surface areas (BET, ASAP 2020, Micromeritics Co., Norcross, GA, USA). The crystal structure of PCN−224 NPs was analyzed by X-ray diffraction patterns (XRD, D8 ADVANCE, Bruker, Karlsruhe, Germany). Optical properties were tested by ultraviolet-visible spectrophotometry (UV-Vis, TU-1901, PERSEE Co., Beijing, China), a steady-state/lifetime spectrofluorometer (QM/TM, Protein Technologies, Inc., Tucson, AZ, USA), and a confocal laser scanning microscope (CLSM, LSM 700, ZEISS, Oberkochen, Germany). The chemical changes of NPs and NFMs were confirmed by a Fourier transform infrared spectrometer (FT-IR, Spectrum Two, PerkinElmer, Waltham, MA, USA). Thermal properties were carried out on thermogravimetric analysis (TGA, TGA 4000, PerkinElmer, Waltham, MA, USA) at a heating rate of 15 °C/min, ranging from 30 °C to 800 °C.

## 3. Results and Discussion

### 3.1. Characteristics of PCN−224 NPs

**Figure 1:**
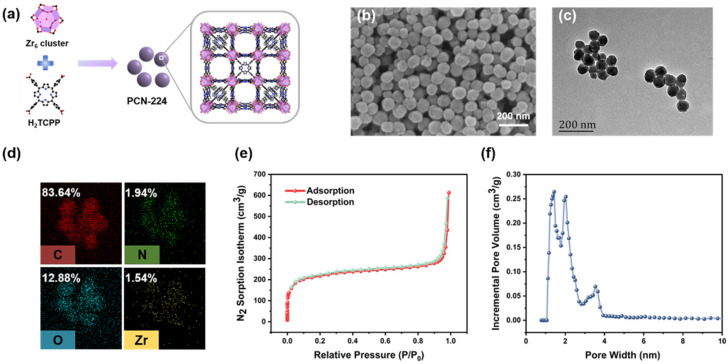
(**a**) The illustration of PCN−224 NPs 3D framework structure. (**b**) The SEM image of PCN−224 NPs. (**c**) The TEM image of PCN−224 NPs and corresponding TEM elemental mappings is shown in (**d**). (**e**) The *N_2_* adsorption-desorption isotherms of PCN−224 NPs, and the corresponding pore size distribution is displayed in (**f**).

In our ‘MOF-first’ strategy, MOFs should be pre-synthesized firstly and then mixed with the polymer solution for fiber processing. As shown in Figure 1a, PCN−224 NPs consist of the six-connected Zr6 clusters and the four carboxyl porphyrin ligand TCPPs through coordination; each Zr6 cluster bridges six TCPP ligands forming the spacious three-dimensional nanochannel framework [52]. The morphological analysis of the pre-synthesized PCN−224 NPs were characterized by SEM and TEM. As shown in Figure 1b,c, PCN−224 NPs present a typical spherical appearance with an average diameter of 79.02 ± 0.58 nm (Figure S1) and good dispersity in the solution. To analyze whether the porphyrins were introduced into the frameworks, the TEM elemental mapping of PCN−224 NPs were examined (Figure 1d and Figure S2). The appearance and even distribution of Zr, O, and N elements, indicating that the existence of porphyrin throughout the whole framework preliminarily [53]. Furthermore, the calculated ratio of Zr and porphyrin in the MOF is about 3.18, which is comparable to the ratio in the theoretical synthetic framework (by the structure of PCN−224, the theoretical ratio of Zr is 4 of that of TCPP) [53,54,55]. Hereafter, *N_2_* adsorption-desorption isotherm measurement was used to reveal the porous structure of the PCN−224 NPs. As demonstrated in Figure 1e, the PCN−224 NPs had a BET surface area of 837.66 m^2^/g, and the pore size distribution is mainly concentrated at 1.43 nm (Figure 1f). It can be seen that the introduction of porphyrin did not sacrifice the porous features of MOFs, which would be conducive to the efficient diffusion of ^1^O_2_ [56,57].

**Figure 2:**
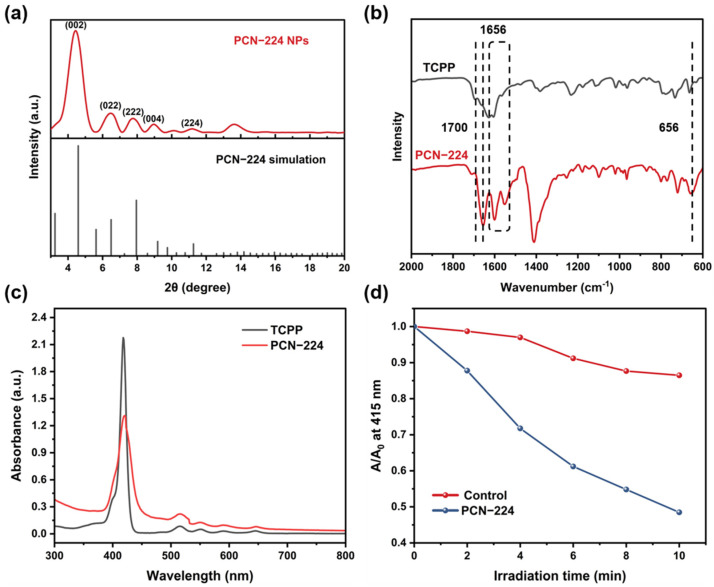
(**a**) Powder X-ray diffraction (PXRD) patterns of PCN−224 NPs. (**b**) The Fourier transform infrared (FT-IR) spectra of PCN−224 NPs and tetrakis (TCPP). (**c**) The ultraviolet (UV)-visible absorption spectra of PCN−224 NPs and N,N-dimethylformamide (DMF) solvent. (The insets showed that the corresponding photographic images of DMF solvent and PCN−224 NPs dispersed in DMF) (**d**) The relative absorbance of DPBF solutions incubated with PCN−224 NPs at 415 nm as a function of irradiation times.

Attempting to identify the crystallographic structure of pre-synthesized PCN−224 NPs, XRD analysis was performed. As shown in Figure 2a, the characteristic diffraction peaks of the synthesized PCN−224 NPs were in excellent agreement with the simulation pattern at the angles of 3° to 20°, proving the crystalline phase purity. The diffraction peaks of PCN−224 NPs at 2θ = 4.46°, 6.46°, 7.78°, 8.98°, and 11.20° which represent the (002), (022), (222), (004) and (224) crystal planes, respectively [58], also indicating that the porphyrin-related inhibition effect on crystal formation was not displayed. In the FTIR spectra (Figure 2b), the peak at 1700 cm^−1^ corresponds to C=O stretching band. The strong vibration assigned to C=O was found in the peak of 1656 cm^−1^, which was owing to the coordination with metal ions [49]. The C=C bond stretching vibration peak of benzene and pyrrole ring appeared at 1552–1601 cm^−^^1^ [59]. Furthermore, it is essential to observe a peak at 658 cm^−1^ which was owing to the Zr–OH bond vibration [49]. The XPS spectrum of Zr 3d (Figure S3) shows that compared with the standard spectrum data (182.4 ev), the Zr 3d_5/2_ peak shifts 0.2 eV toward the lower binding energy. This shift indicates the charge redistribution within Zr6 nodes in PCN−224 NPs, suggesting the successful coordination of the carboxyl group of the porphyrin to unsaturated sites of Zr6 [60]. The successful synthesis of PCN−224 NPs was demonstrated by the above results.

### 3.2. Studying Activity of PCN−224 NPs

With an indication of the porphyrin introduction, the question remained as to whether the porphyrin retained its photodynamic functionality. We thus carried out the UV-Vis analysis to characterization PCN−224 NPs. As illustrated in Figure 2c, PCN−224 NPs demonstrated the main absorption peak (Soret band) at 420 nm and four weak absorption peaks (Q bands) in a range of 500–700 nm, similar to the TCPP spectrum. The Q bands of TCPP were indicated that the Zr ions were not coordinated to the center of the porphyrin ligands [61]. In contrast, the Soret band of PCN−224 is significantly weaker and demonstrates a slight redshift, which may be due to ligand-to-metal charge transfer caused by the strong coordination of the TCPP linkers to the Zr-O clusters in the PCN−224 framework [62,63]. The photoluminescence (PL) spectra show two strong emission bands of PCN−224 NPs at about 658 nm and 721 nm under an excitation wavelength of 430 nm, which are typical emission peaks for the transition of porphyrins from S_1_→S_0_ states, corresponding to Q (0-0) and Q (0-1) transitions, respectively (Figure S4) [64,65]. This absorption and PL spectra pattern are the well-known characteristic of the monomeric porphyrin molecules [66]. This demonstrates the structural integrity of the porphyrin ligand in the PCN−224 NPs framework, while showing as well that red fluorescence can be used as an imaging label for PCN−224 NPs [49]. In addition, the PL spectrum underwent a similar change to the UV-Vis. This may be caused by the coordination of the TCPP ligand to the Zr6 cluster changing the molecular planarity in the porphyrin macrocycle and affecting the distribution of the porphyrin electron density [63,67].

^1^O_2_ is the most destructive bacterial substance in ROS. To further investigate the PDT activities of PCN−224 NPs, the generation capabilities of ^1^O_2_ from PCN−224 NPs under a visible light lamb were measured by using the DPBF probe. As depicted in Figure 2d and Figure S5, the absorbance at 415 nm of pure DPBF solution displayed a slight decrease after 10 min of irradiation. This phenomenon is due to the self-decomposition of DPBF since it is extremely sensitive to external light [39]. By contrast, the absorbance of the DPBF solution with the addition of PCN−224 NPs has significant decay trends in the same conditions, demonstrating their ^1^O_2_ generation capability. These results prove the effectiveness of the one-step synthesis route for the preparation of PCN−224 NPs, which have important potential as a functional photosensitizer carrier and PDT agent for the treatment of bacterial infections.

### 3.3. Nanofiber Fabrication with Preformed PCN−224

**Figure 3:**
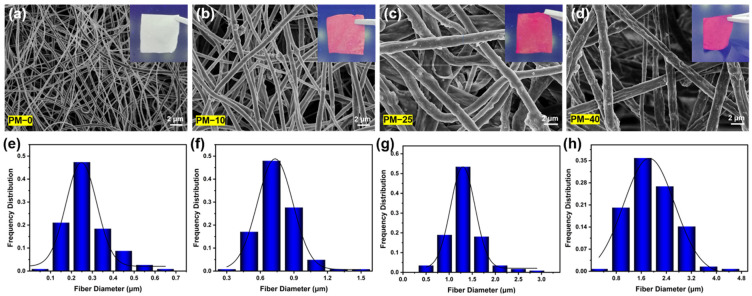
(**a**–**d**) Scanning electron microscope (SEM) images of PM−0, PM−10, PM−25, and PM−40 NFMs, the inset show the Photos of corresponding NFMs under UV lamp irradiation. (**e**–**h**) The corresponding diameter distributions of various membranes (*n* = 100).

Co-electrospinning offers a facile route for loading MOFs in polymeric fibers. Low MOFs loadings can facilitate the maintenance of the structural integrity of the NFMs morphology and three-dimensional porous structure, however, there have been examples of NFMs, that exhibit improved antibacterial and gas adsorption performance when the MOFs content in the NFMs was increased [39,68]. Meanwhile, the chemical differences between MOFs crystals and polymers can manifest as defects (such as stability, thermal stability, etc.) in the composite NFMs [69]. Accordingly, we use different concentrations of PCN−224 NPs to fabricate NFMs. The surface morphology of the nanofibers after assembly with preformed PCN−224 NPs was observed in Figure 3. As shown in Figure 3a–d, the surface of neat PCL nanofibers (PM−0) was smooth, whereas after PCN−224 NPs were introduced, the surface morphology of nanofibers changed obviously. Compared with pristine PM−0 nanofibers, only a few PCN−224 NPs were sporadically distributed on the surface of PM−10 nanofibers, which due to the lowest loading amount of PCN−224 NPs and the NPs are mainly distributed in the fiber matrix. It is noteworthy that as the concentration of the PCN−224 NPs increases, the NPs on the surface of the nanofibers were sharply increased, and even formed agglomerates, resulting in the PM−25 and PM−40 nanofibers showing a significantly rougher surface morphology. Meanwhile, it is observed that a dramatic increase in the nanofiber diameter from 0.25 to 1.85 μm with the gradual increment in the dosage of PCN−224 NPs (Figure 3e–h). This phenomenon may be ascribed to the enhanced viscosity of the spinning solution with the incorporation of PCN−224 NPs, which prolongs the relaxation time and restricts the motion of the polymer chains [32].

**Figure 4:**
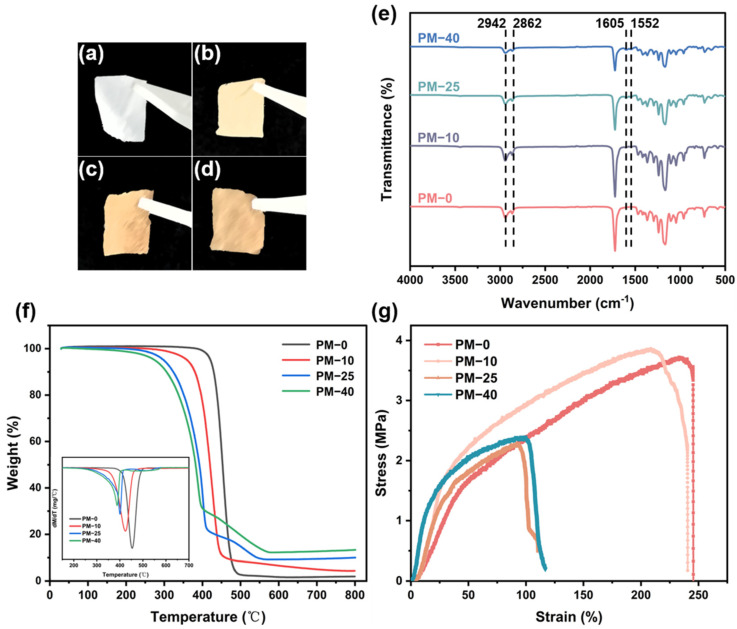
Photographs of the PM−0 (**a**), PM−10 (**b**), PM−25 (**c**), and PM−40 (**d**) NFMs. (**e**) FTIR spectra of PM−0, PM−10, PM−25, and PM−40 NFMs. (**f**) Thermogravimetric analysis (TGA) curves of nanocomposite fibrous membranes, the inset show the corresponding DTG analysis of PM−0, PM−10, PM−25, and PM−40 NFMs. (**g**) The mechanical properties of PM−0, PM−10, PM−25, and PM−40 NFMs.

Furthermore, the successful loading of PCN−224 can also be verified by the significant color changes of PM NFMs as displayed in Figure 4a–d. The initial color of PM−0 NFMs appears white, while the color of the NFMs with PCN−224 NPs changes into brown-red. Comparing the FTIR spectra of PM NFMs in Figure 4e, it can be found that the asymmetric and symmetric vibrations of CH_2_ at 2942 cm^−1^ and 2862 cm^−1^ were ascribed to PM−0 and the C=C bond stretching vibration appeared at 1552–1605 cm^−1^, which was attributed to the presence of PCN−224 NPs [70]. The FTIR results indicating that the PCN−224 NPs were successfully introduced into PCL nanofibers [58,71]. The DSC results (Figure S6) demonstrate the possible coordination interaction between Zr metal nodes and PCL carbonyl groups under the introduction of PCN−224 [72,73]. Nevertheless, as a PDT wound dressing, the stable integration of PS NPs and nanofibers is the most staple requirement. For this reason, we immersed the PM NFMs in HEPES buffer (50 mM, pH = 7.4) solution and tested the UV-visible spectrum of the soaking solution to explore the stability of the PM NFMs (Figure S7). The results showed that the curves of PM NFMs are completely consistent, and no characteristic peaks of PCN−224 NPs were detected, which indicates that no PCN−224 NPs were shed from the fibers and they are stably and firmly combined with PCL nanofibers. In addition, the results (Figure S8) of XRD show that the PM−0 NFMs have two diffraction peaks at 21.5° and 23.8°, which are characteristic peaks of neat PCL, corresponding to the (110) and (200) planes, respectively [74]. The PM−25 NFMs retain the characteristic diffraction peaks of neat PCL, while the characteristic diffraction peaks of PCN−224 appear. This indicates that the structure of PCN−224 NPs in the composite membrane has been well preserved.

Accurate knowledge of the PCN−224 NPs loading during electrospinning is important because excess PCN−224 NPs in the polymeric solution can result in needle clogging and instability of the jets. Therefore, the TGA test was used to determine the actual loading of PCN−224 NPs in NFMs (Figure 4f). It can be clearly observed that the residual mass fraction of NFMs at 800 °C shows an increasing trend with the increase of PCN−224 NPs content. Subsequently, the calculation was performed according to previous research [75,76], and it was assumed that the only substance present at 800 °C is the secondary building unit (ZrO_2_) derived from PCN−224 NPs; the effective loading rate of PCN−224 NPs in the NFMs was deduced by a comparison of the residual mass fraction of ZrO_2_ of these NFMs. The residual mass fraction of ZrO_2_ in PM−10 NFMs was about 4.48 wt%. Using this as a standard, the theoretical residual mass fractions of PM−25 and PM−40 NFMs were 10.95 wt% and 17.52 wt%, respectively. However, the test results showed that the actual residual mass fraction of 10.00 wt% for PM−25 NFMs and 13.32 wt% for PM−40 NFMs. It can be seen that there exist certain differences in theoretical and practical residuals, especially in PM−40 NFMs. This indicates that the actual loading efficiency of PCN−224 NPs in the PM−40 NFMs is low. This phenomenon was to be anticipated because excessive PCN−224 NPs will form agglomerates that are unevenly dispersed in the polymeric solution, and clog the needles during the electrospinning process, resulting in the difficult formation and poor consistent of fibers, which can also be observed in the above SEM images. Furthermore, the DTG curves revealed that although the introduction of PCN−224 NPs affects the crystal structure of the PCL polymers, the thermal degradation temperature of NFMs decreased slightly from 450 °C to 390 °C with the increase in loading amount, but its thermostability still met the requirements of practical applications.

In addition, the tensile strengths of the PM NFMs were measured to evaluate the influence of the PCN−224 NPs on the mechanical properties of PM NFMs. As shown in Figure 4g, the neat PM−0 NFM showed the highest mechanical breaking elongation of 245.45% with breaking strength at 3.72 MPa. Slightly increased breaking strength (3.86 MPa) and decreased breaking elongation (240.55%) could be observed in PM−10 NFMs. This phenomenon may be due to the coordination interaction between the carbonyl group of PCL and the Zr metal nodes of PCN−224, and the more uniform distribution of PCN−224 NPs without forming obvious weak defects, thus improving the tensile strength of the membranes to a certain extent [72,77]. After more PCN−224 NPs were introduced, the breaking strength and breaking elongation of PM−25 and PM−40 NFMs were decreased to a large extent. This is attributed to the agglomerate of PCN−224 NPs in the membranes to form stress concentration centers, which increases the weak defects. Moreover, due to the increase in PCN−224 NPs concentration, the spinnability of the nanofiber is reduced, resulting in a decrease in the uniformity of the nanofiber [77,78].

### 3.4. ^1^O_2_ Generation of PM NFMs

**Figure 5:**
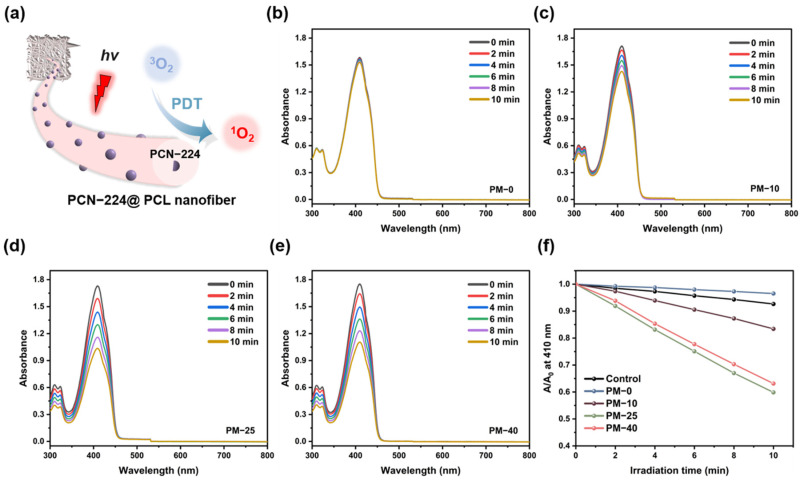
(**a**) The generation mechanism schematic of ^1^O_2_ by membranes with PCN−224 NPs introduced. UV-visible spectrum of 1,3-diphenylisobenzofuran (DPBF) under visible illumination (630 nm, 100 mW/cm^2^) in the present of (**b**) PM−0, (**c**) PM−10, (**d**) PM−25 and (**e**) PM−40 NFMs. (**f**) The relative absorbance of DPBF solutions was incubated with the membranes at 410 nm as a function of irradiation time.

As a proof of concept, we also demonstrated the ^1^O_2_ generation ability of PM NFMs. The mechanism of PM NFMs in the generation of ^1^O_2_ is summarized in Figure 5a. Under the excitation of the laser with appropriate wavelength, PCN−224 NPs, as a new PS, can react with ^3^O_2_ through energy transfer to generate cytotoxic ^1^O_2_, which can kill bacteria by attacking the bacterial biomolecules [56]. The results of employing the sensing probe DPBF to investigate the capacities of PM NFMs to generate ^1^O_2_ are shown in Figure 5b–f and Figure S9, after 10 min of visible light irradiation (630 nm, 100 mW/cm^2^), the characteristic absorption at 410 nm of pure DPBF solution and the solution incubated with the PM−0 NFMs decreased slightly. This phenomenon is attributed to the fact that DPBF is sensitive to light and oxygen in the air, and produces a negligible amount of ^1^O_2_. While the characteristic absorption intensity of DPBF solution incubated with the PM−10, PM−25, and PM−40 NFMs decreased rapidly with continuous irradiation. The relative absorbance of DPBF solutions incubated with PM−25 and PM−40 NFMs are much smaller than that of PM−10. These results indicate that the amount of ^1^O_2_ generated from PM NFMs is strongly associated with the introduced content of PCN−224 NPs. It is worth noting that the ^1^O_2_ generation capacity of PM−25 NFMs is superior to PM−40 NFMs, even though the PCN−224 loading amount of PM−40 NFMs is higher.

### 3.5. The Morphologies and Distribution of PCN−224 NPs in PM NFMs

**Figure 6:**
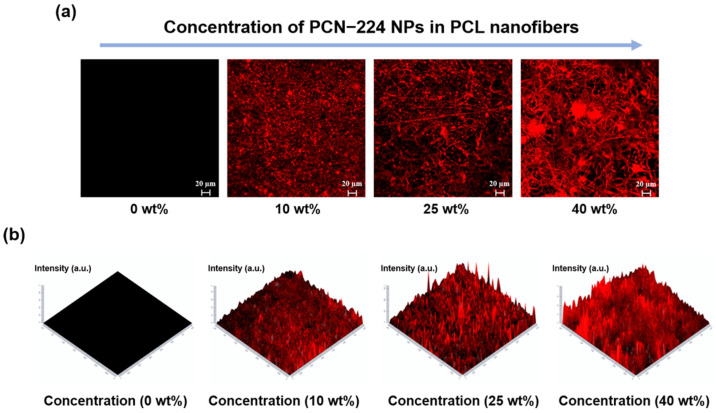
(**a**) The confocal laser scanning microscope (CLSM) images of various PM membranes with different PCN−224 NPs content and (**b**) their normalized fluorescence intensity and distribution (λ_ex_ = 430 nm).

To further clarify the morphologies and distributions of PCN−224 NPs in the nanofibers, the CLSM images of each NFMs were measured. As shown in Figure 6a, upon 430 nm laser excitation, the nanofibers loaded with PCN−224 NPs showed obvious red fluorescence, indicating that PCN−224 NPs were stably distributed in the nanofibers. The fluorescence images with distinct and continuous fibrous outlines were observed with the increased PCN−224 NP content, which is consistent with the fiber diameter distribution results. Moreover, the significant agglomerations of PCN−224 NPs can be observed on the PM−40 NFMs compared with the fluorescence images of PM−25 and PM−10 NFMs. This type of morphology is typical, such that the resultant NFMs are non-uniform with unpredictable properties, which is consistent with the results of the ^1^O_2_ generation performance. It can be observed from Figure 6b that the red fluorescence signal of NFMs was enhanced with the content of PCN−224 NPs increases. These results powerfully confirmed that the distribution and amount of PCN−224 NPs in the membrane affect its ability to generate ^1^O_2_.

### 3.6. In Vitro Antibacterial Activity Assay

**Figure 7:**
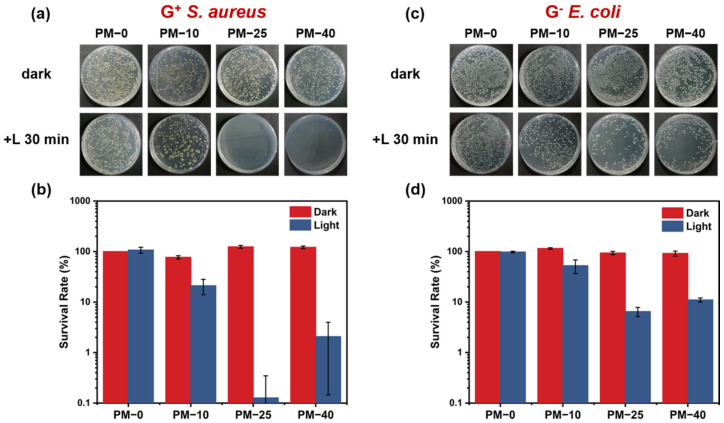
Photographs of residual *S. aureus* (**a**) and *E. coli* (**c**) bacterial colonies under various membranes treatments in the dark/light. *S. aureus* (**b**) and *E. coli* (**d**) relative bacterial survival rate. The error bars indicate the standard deviation (*n* = 3).

Gram-positive *S. aureus*, Gram-negative *E. coli*, and drug-resistant bacteria *MRSA* were selected as representative strains, and the photodynamic antibacterial performance of the PM NFMs were evaluated by the method of counting live bacteria on the plate. Initially, the antibacterial ability of PM NFMs was visually and qualitatively evaluated through monitoring the photos of the agar plate coated with the residual bacteria after PM NFMs treatment (Figure 7a,c), respectively. In the dark, all membranes groups showed densely distributed colonies on the agar plate, indicating that all the membranes had no antibacterial activity in the darkest condition. In contrast, the membranes introduced with PCN−224 NPs under light irradiation exhibited efficient photodynamic antibacterial effects on *S. aureus* and *E. coli*, as indicated by the sparsely distributed bacterial colonies. Furthermore, the survival rate of bacteria was calculated to quantitatively evaluate the antibacterial activity of the PM NFMs (Figure 7b,d). Compared with PM−0 NFMs, the PM−10 NFMs exhibited slightly antibacterial activity against *S. aureus* and *E. coli* within 30 min light irradiation due to the lowest loading amount of PCN−224 NPs. The corresponding survival rates of 21.04% for *S. aureus* and 52.51% for *E. coli*. An improvement in antibacterial activity was noted as the increased concentration of PCN−224 NPs contained in the membranes. In detail, the PM−25 NFMs exhibited excellent potent antibacterial properties with survival rates of 0.13% for *S. aureus* and 2.06% for *E. coli* upon 30 min irradiation, respectively. For the PM−40 NFMs, the survival rates of *S. aureus* and *E. coli* are 6.48% and 11.04% under 30 min irradiation, respectively. It could be clearly observed that the antibacterial ability of PM−25 NFMs was superior to the PM−40 NFMs, which was in accordance with the previous result of ^1^O_2_ generation. These results confirmed that the prepared membranes containing PCN−224 NPs have significant antibacterial activities, and also demonstrated that the antibacterial activity is strongly associated with the loading dose and distribution of PCN−224 NPs. The bacteria were attached to the surface of the composite membrane, and the nanofiber morphology did not change significantly under the dark/light treatment, which indicated that the bacteria would not affect the nanofiber structure (Figure S10). It is also worth mentioning that the different sensitivities to ^1^O_2_ between Gram-positive *S. aureus* and Gram-negative *E. coli*. The Gram-negative bacteria showed more tolerance to ^1^O_2_ than Gram-positive bacteria, which is ascribed to the different structures of bacterial cell walls. The Gram-negative bacteria contain an additional layer of lipopolysaccharide on the outside of the peptidoglycan layer, which has a high degree of impermeable, making it difficult for ^1^O_2_ to enter the inside of the bacteria, and cannot destroy bacterial biomolecules such as proteins, nucleic acid and lipids [58].

MRSA is a typical example, it has become one of the most prevalent pathogens in clinic wound infections [79,80,81]. The local skin infection caused by it is almost resistant to all conventional antibiotics, greatly increases the difficulty of clinical treatment, and seriously threatens public health [82]. Hence, MRSA was selected as the representative strain of drug-resistant bacteria to evaluate the performance of PM NFMs in eliminating drug-resistant bacteria (Figure S11). The results show that the PM−25 NFMs exhibited excellent antibacterial properties with survival rates of 1.91% for *MRSA* upon 30 min irradiation. The antibacterial test results show that the photodynamic composite membranes prepared by introducing PCN−224 NPs can effectively eliminate drug-resistant bacteria and common bacteria that are widely present in the wound, and has excellent antibacterial effects.

### 3.7. Cytotoxicity Assay

**Figure 8:**
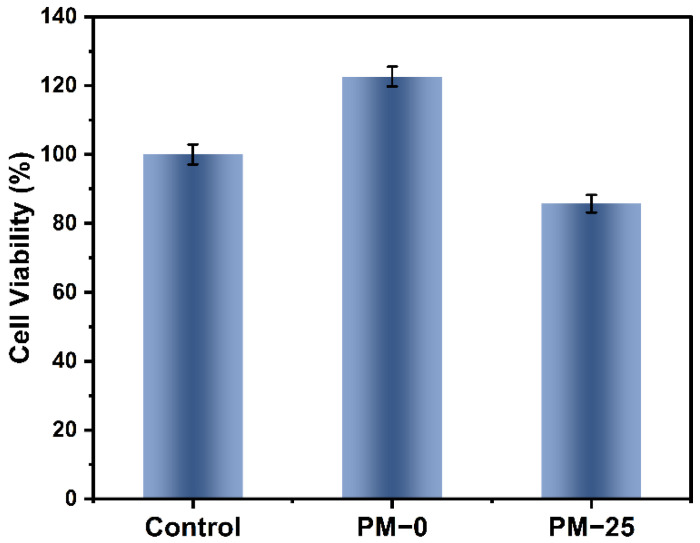
Cell viability of L929 fibroblasts on membranes of PM−0 and PM−25 NFMs (*n* = 3).

Last but not least, we used the CCK-8 method to measure the viability of L929 cells co-cultured with PM−0 and PM−25 NFMs for 24 h to evaluate the cytotoxicity of the composite membrane. As shown in Figure 8, the viability of the cells on the PM−0 NFMs reached 123%, indicating that the neat PCL membrane has excellent biocompatibility, which is consistent with the results of previous studies. The cells co-cultured with PM−25 NFMs also maintained high viability (85%), which indicates that the nanocomposite membrane after the introduction of PCN−224 NPs has good biocompatibility, which is beneficial for its application in anti-infection and wound healing.

## 4. Conclusions

In conclusion, we adopted the ‘MOF-first’ strategy to pre-synthesize porphyrinic MOFs nanoparticles (PCN−224 NPs) firstly, then mixed with the polymer solution to fiber processing via one-step co-electrospinning technology, and successfully developed PM NFMs with PDT antibacterial properties. The structure of PCN−224 NPs can be well preserved after integration with PCL, and a large number of firm loads can be achieved on the nanofibers. Both PCN−224 NPs and PM NFMs showed excellent ability of ^1^O_2_ generation. The PM NFMs could increase the ^1^O_2_ generation and exhibit excellent PDT antibacterial against *S. aureus*, *E. coli*, and *MRSA* in vitro based on the increased loading of PCN−224 NPs. Importantly, the PM−25 NFMs had the best PDT antibacterial performance with survival rates of 0.13% for *S. aureus*, 2.06% for *E. coli*, and 1.91% for *MRSA* upon 30 min irradiation, due to a homogeneous and stable loading of PCN−224 NPs in nanofibers. The cytotoxicity assay verified that the PM NFMs possessed good biocompatibility. Taken together, these NFMs functionalized by porphyrinic MOFs could be regarded as a promising wound dressing, and could be widely applied in the field of wound antibacterial infection.

## Data Availability

The data presented in this study are available on request from the corresponding author. The data are not publicly available due to privacy or ethical restrictions.

## Supplementary Materials

The following are available online at https://www.mdpi.com/article/10.3390/polym13223942/s1, Figure S1: The size distribution of the PCN−224 NPs, Figure S2: The transmission electron microscopy (TEM) elemental mapping results of PCN−224 NPs, Figure S3: XPS spectra of PNC-224 and Zr 3d, Figure S4: The PL spectra of PCN−224 NPs, Figure S5: UV-visible spectrum of DPBF under visible illumination (630 nm, 100 mW/cm^2^) with/without PCN−224 NPs, Figure S6. DSC scans of PM−0 and PM−25 NFMs. Left) heating and Right) cooling, Figure S7: The UV-visible spectrum of PM NFMs after immersing in HEPES buffer for 12 h, Figure S8: XRD patterns of PM−0 and PM−25 NFMs, Figure S9: UV-visible spectrum of DPBF under visible illumination (630 nm, 100 mW/cm^2^), Figure S10. The SEM images of bacteria and bacteria treated fibers, Figure S11: Relative bacterial survival rate and photographs of residual colonies of MRSA under various membranes treatments in the dark/light.
