# Supplementary material for: Integrating Porphyrinic Metal-Organic Frameworks in Nanofibrous Carrier for Photodynamic Antimicrobial Application
Source: Polymers (Basel). 2021 Nov 15;13(22):3942. doi: 10.3390/polym13223942 (PMC8625335; doi:10.3390/polym13223942)
Supplement: Supplementary file 1 [file polymers-13-03942-s001.zip › polymers-1439776-supplementary.pdf]

polymers

Supplementary Material

Integrating Porphyrinic Metal-Organic Frameworks in Nanofibrous Carrier for Photodynamic Antibacterial Application

Huiru Zhang [1,2], Zhihao Xu [1,2], Ying Mao [1,2], Yingjie Zhang [1,2], Yan Li [1,2,*], Jihong Lao [1,2], and Lu Wang [1,2]

1 Key Laboratory of Textile Science &Technology, Ministry of Education, College of Textiles, Donghua University, Shanghai 201620, China; zhrlzc@163.com (H.Z.); lyabblove@126.com (Z.X.); maoying_dhu@163.com (Y.M.); 15900238890@163.com (Y.Z.); ljh@dhu.edu.cn (J.L.); wanglu@dhu.edu.cn (L.W.)
2 Key Laboratory of Textile Industry for Biomedical Textile Materials and Technology, Donghua University, Shanghai 201620, China;
* Correspondence: yanli@dhu.edu.cn; Tel.: +86-21-6779-2634

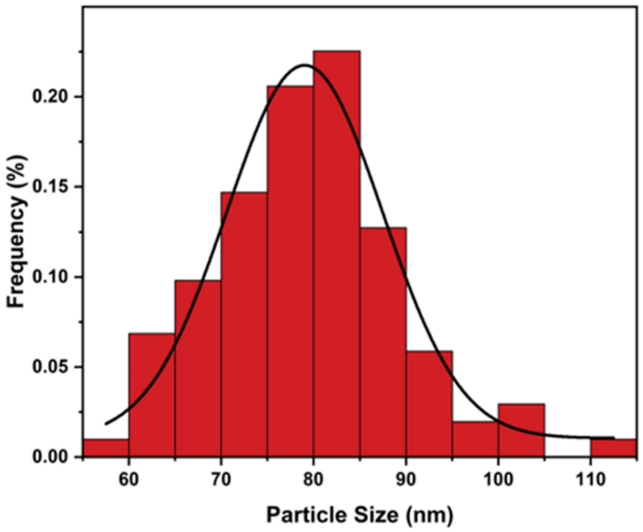

Figure S1. The size distribution of the PCN−224 NPs.

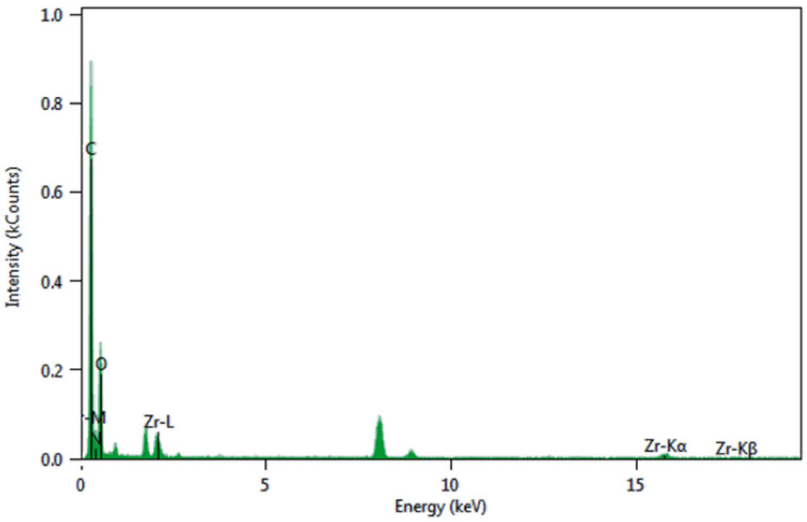

Figure S2. The TEM elemental mappings results of PCN−224 NPs.

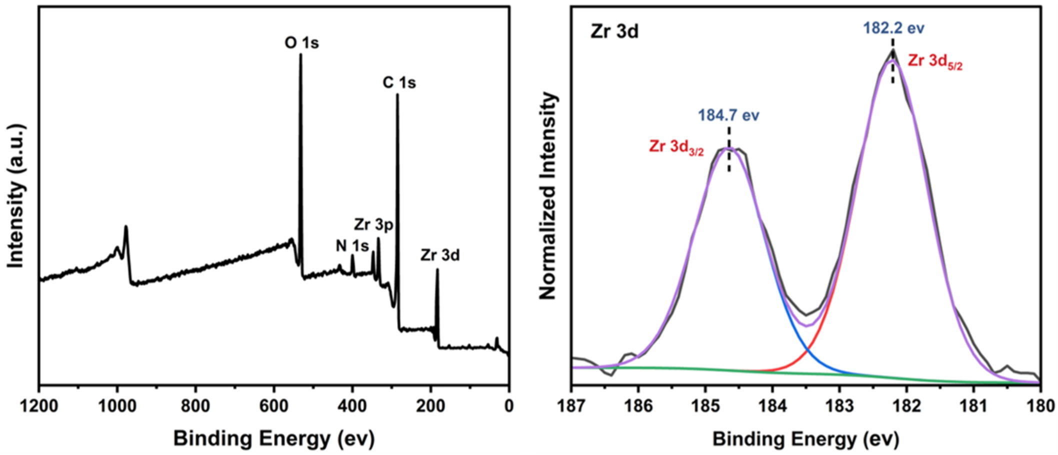

Figure S3. XPS spectra of PCN−224 and Zr 3d.

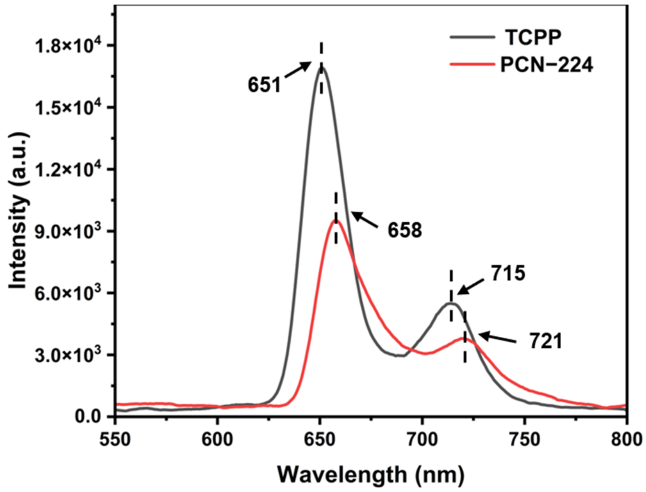

Figure S4. The PL spectra of PCN−224 NPs.

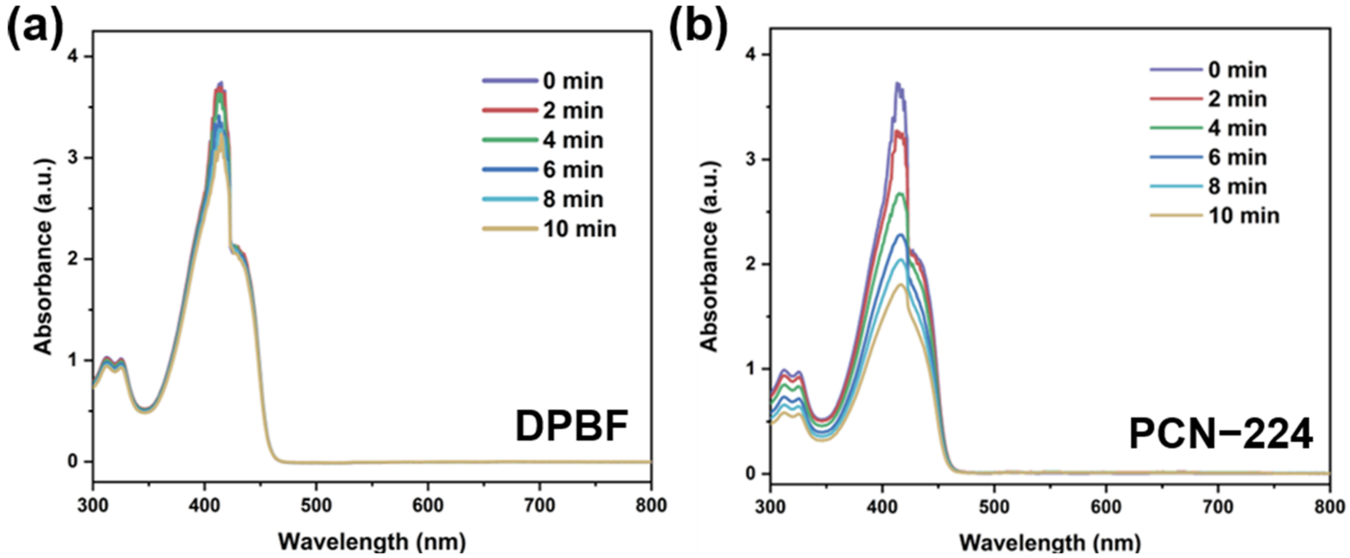

Figure S5. UV-visible spectrum of DPBF under visible illumination (630 nm, 100 mW/cm^2) with/without PCN−224 NPs.

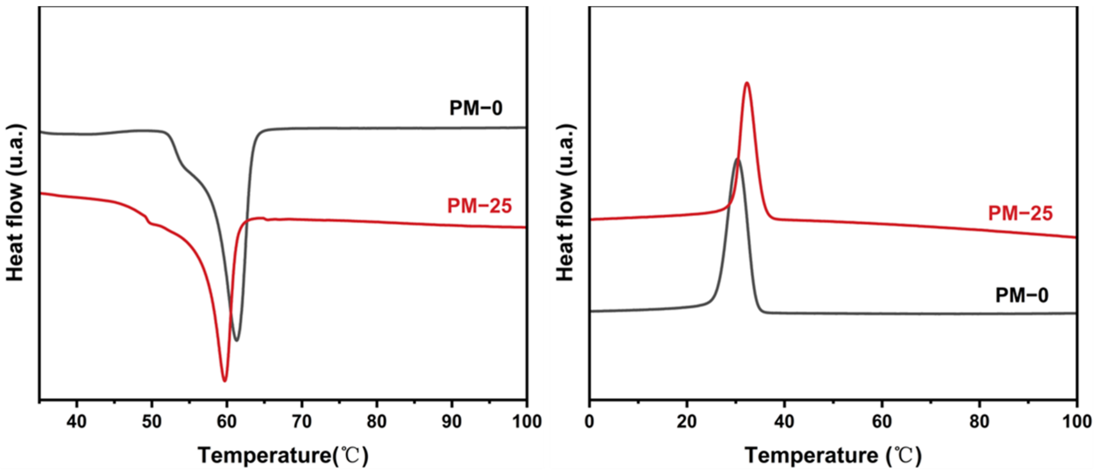

Figure S6. DSC scans of PM−0 and PM−25 NFMs. Left) heating and Right) cooling.

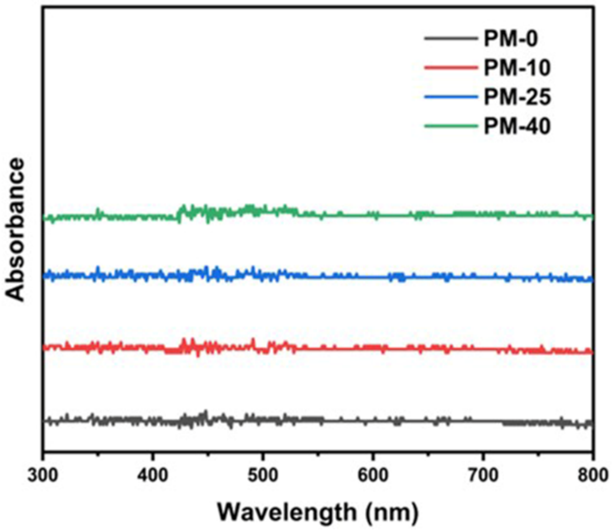

Figure S7. The UV-visible spectrum of PM NFMs after immersing in HEPES buffer for 12 hours.

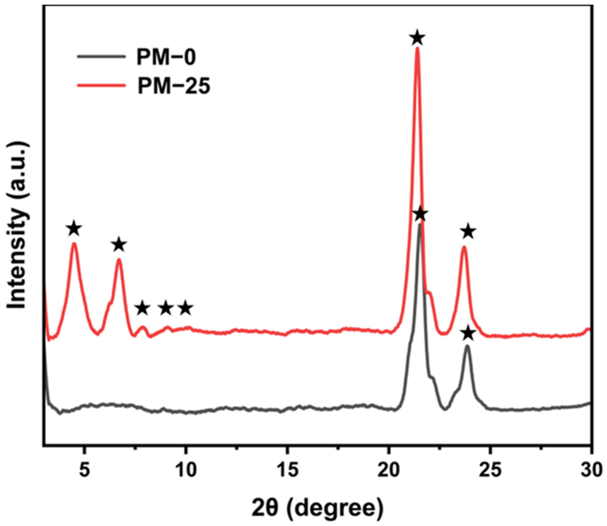

Figure S8. XRD patterns of PM−0 and PM−25 NFMs

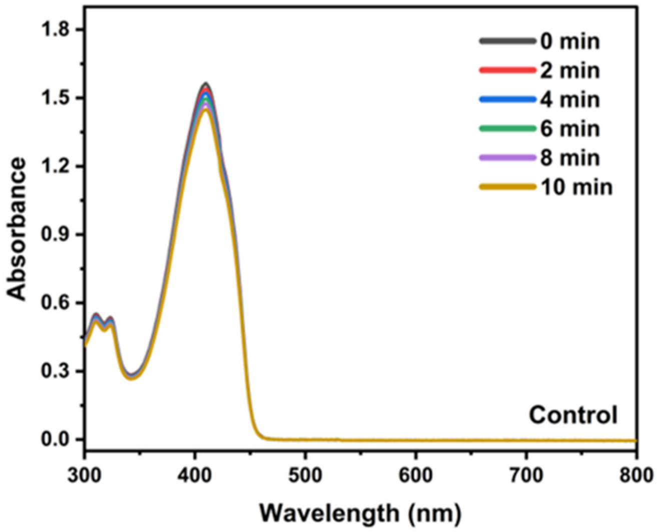

Figure S9. UV-visible spectrum of DPBF under visible illumination (630 nm, 100 mW/cm^2).

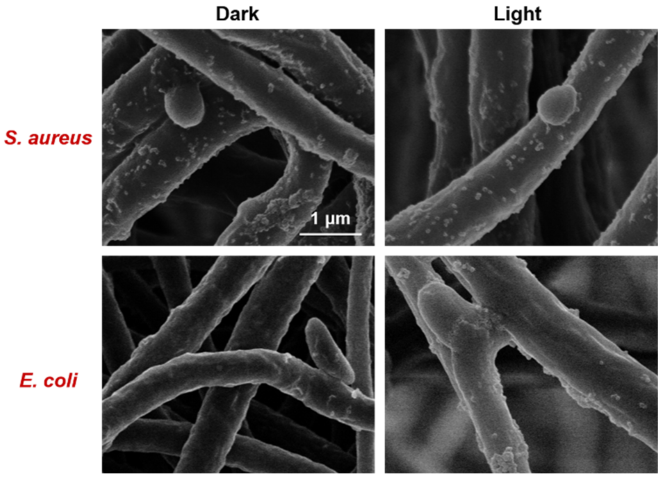

Figure S10. The SEM images of bacteria and bacteria treated fibers.

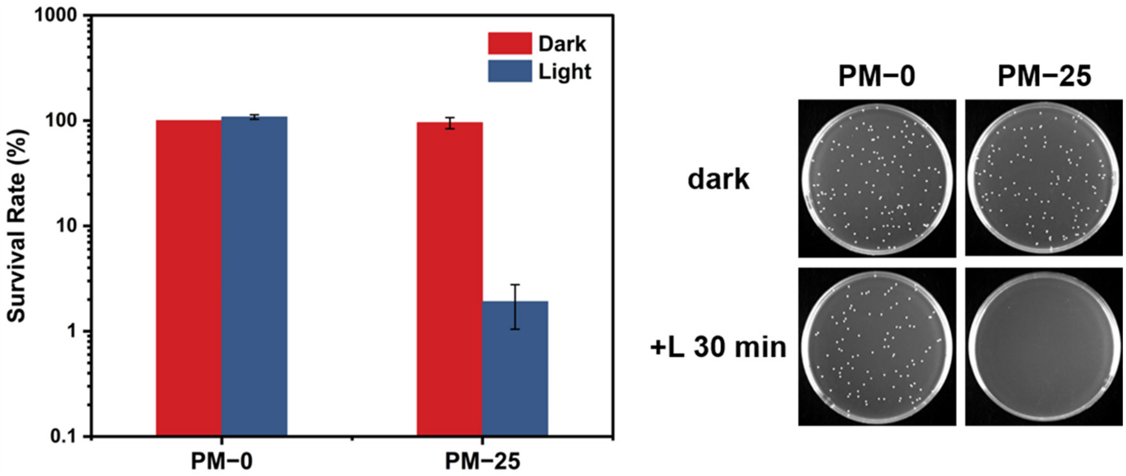

Figure S11. Relative bacterial survival rate and photographs of residual colonies of MRSA under various membranes treatments in the dark/light. The error bars indicate the standard deviation (n=3).
